# Neurotherapy for chronic headache following traumatic brain injury

**DOI:** 10.1186/s40779-015-0049-y

**Published:** 2015-08-31

**Authors:** David V. Nelson, Mary Lee Esty

**Affiliations:** Department of Psychology and Philosophy, Sam Houston State University, Box 2447, Huntsville, TX 77341 USA; Brain Wellness and Biofeedback Center of Washington, 7910 Woodmont Ave #305, Bethesda, MD 20814 USA

**Keywords:** Chronic headache, Posttraumatic headache, Traumatic brain injury, Neurotherapy, Electroencephalograph (EEG) biofeedback, Neurofeedback

## Abstract

**Background:**

Chronic headache following traumatic brain injury (TBI) sustained in military service, while common, is highly challenging to treat with existing pharmacologic and non-pharmacologic interventions and may be complicated by co-morbid posttraumatic stress. Recently, a novel form of brainwave-based intervention known as the Flexyx Neurotherapy System (FNS) that involves minute pulses of electromagnetic energy stimulation of brainwave activity has been suggested as a means to address symptoms of TBI. This study reports on a clinical series of patients with chronic headache following service-connected TBI treated with FNS.

**Methods:**

Nine veterans of the wars in Afghanistan and Iraq with moderate to severe chronic headaches following service-connected TBI and complicated by posttraumatic stress symptoms were treated in 20 individual FNS sessions at the Brain Wellness and Biofeedback Center of Washington (in Bethesda, Maryland, USA). They periodically completed measures including the Brief Pain Inventory-Headache (BPI-HA) past week worst and average pain ratings, the Posttraumatic Stress Disorder Checklist-Military version (PCL-M), and individual treatment session numerical rating scale (NRS) for degree of cognitive dysfunction. Data analyses included beginning to end of treatment t-test comparisons for the BPI-HA, PCL-M, and cognitive dysfunction NRS.

**Results:**

All beginning to end of treatment t-test comparisons for the BPI-HA, PCL-M, and cognitive dysfunction NRS indicated statistically significant decreases. All but one participant experienced reduction in headaches along with reductions in posttraumatic stress and perceived cognitive dysfunction, with a subset experiencing virtual elimination of headaches. One participant obtained modest headache relief but no improvement in posttraumatic stress or cognitive dysfunction.

**Conclusions:**

FNS may be a potentially efficacious treatment for chronic posttraumatic headache sustained in military service. Further research is needed to investigate the efficacy of FNS within a randomized, controlled clinical trial, to identify characteristics of those most likely to respond, and to explore underlying mechanisms that may contribute to improvement.

## Background

Traumatic brain injury (TBI), especially mild TBI (mTBI), has become the “signature injury” of recent warfare, including the involvement of military service personnel in the wars in Afghanistan and Iraq [1]. Among the leading factors contributing to this are various mechanisms of concussion and exposure to explosive blasts [1–3]. It has been estimated that from 30 to 90 % of individuals with TBI may develop some form of headache [4]. In one cohort of US military service members returning from Afghanistan or Iraq, 19.6 % met criteria for a deployment-related concussion; among those, 97.8 % reported having headaches during the final 3 months of deployment; and more than 1 in 3 met criteria for posttraumatic headache, that is headache with onset within 7 days after head trauma (or regaining consciousness following head trauma) [5]. Such headaches have been associated with greater frequency of headache attacks and increased prevalence of chronic daily headache [5, 6]. Some evidence suggests mTBI may result in more severe headache patterns relative to more severe cases of TBI, but this remains controversial [7, 8]. In any event, chronic daily headache is a persistent and potentially debilitating sequela of military service related TBI.

Many returning service personnel also experience posttraumatic stress disorder (PTSD) [9]. Although not included in the diagnostic criteria for PTSD, individuals with PTSD are at increased risk for experiencing chronic headaches [1, 10]. Further, symptoms of TBI and PTSD often overlap and/or co-occur in the same individual [11, 12]. There is some evidence suggesting the combination of the two may be associated with overall more severe symptom presentations across shared symptom domains [10, 13]. There is also evidence to suggest TBI and PTSD may share some underlying mechanisms [10, 11]. Furthermore, treatment for chronic headache is complicated by the presence of co-morbid PTSD [8, 10].

Treatment of chronic headache associated with TBI remains highly challenging. The forms of headache most typically manifesting as chronic posttraumatic headache correspond closely to symptom patterns seen in migraine and tension-type headaches [4, 7]. Treatment recommendations typically are extrapolated from the broader headache literature on these more common forms of headache [4, 7, 14]. However, despite typical pharmacologic and non-pharmacologic interventions, many individuals remain highly symptomatic and are vexing to clinicians [8]. While relaxation-based (surface electromyography or temperature/thermal) biofeedback has demonstrated some efficacy in treating the more common types of headaches in the general population, it is far from universally effective [15]. Further, variations of biofeedback incorporating electroencephalograph (EEG) information have also been suggested [16]. However, EEG biofeedback (sometimes also known as neurofeedback or neurotherapy) requires considerable time and effort on the part of both patient and clinician in order for the patient to learn voluntary control over the production or inhibition of specific EEG frequencies or frequency ranges [17].

Recently, a form of brainwave-based intervention known as the Flexyx Neurotherapy System (FNS) has been suggested for addressing mixed symptom presentations of TBI and PTSD [18]. As a novel variant of EEG biofeedback, FNS falls within the bio-energy domain of complementary and alternative medicine. Rather than requiring patients to acquire skills to change patterns of EEG activity, FNS instead involves off-setting stimulation of brainwave activity by means of an external energy source, specifically, the conduction of electromagnetic energy (EM) stimulation via the connecting EEG cables [19]. FNS has been further adapted by utilization of two-channel, versus one-channel only, EEG monitoring. The procedures also do not require active effort on the part of the patients; rather, patients remain relatively passive while their EEG activity is monitored and the information obtained interacts with parameters pre-set within the software to initiate subtle, minute pulses of EM stimulation. The present investigation focuses on a subset of service members from a larger investigation who were experiencing chronic daily headaches after sustaining service-related TBI and for whom the majority also had PTSD. These individuals were all highly symptomatic and had not benefited significantly from typical pharmacologic or psychotherapy interventions.

## Methods

### Institutional Review Board Approval

All methods, including the procedures described below, received appropriate institutional review board approvals from the Committee for the Protection of Human Subjects at Sam Houston State University and the Chesapeake Institutional Review Board, Inc., for the Brain Wellness and Biofeedback Center of Washington. Signed informed consent was obtained from all participants.

### Participants

Participants included 9 (8 male, 1 female) US veterans who had experienced wartime deployments in Afghanistan and/or Iraq. All had experienced service-connected TBI with the majority also having experienced some loss of consciousness (range: a few seconds to a number of minutes). While one participant reported having experienced only one concussion, the others reported having experienced multiple (typically “many” or “too many to count”) traumatic head injuries, including exposures to explosive blasts. Age (in years) ranged from 25 to 64 (M = 37.33, SD = 12.63). The duration since end of their most recent deployment to first treatment in this study ranged from 6 to 103 months, with a median of 46 months. Accordingly, many months following their return from deployment, all were experiencing persistent moderate to severe daily headaches. The majority (8) had been diagnosed to have co-morbid PTSD. Three had been diagnosed with depressive disorders. Two were not taking any prescribed medications at the time of study entry, while the remainder were taking at least one medication (range: 1–7, median 2), including acetaminophen (2 participants), antidepressants (4 participants), anti-anxiety drugs and hypnotics (3 participants), anticonvulsants (2 participants), antihistamines (2 participants), as well as statin (1 participant), alpha-blocker (1 participant), beta-blocker (1 participant), and stimulant (1 participant) drugs; one participant was using a triptan on an as needed basis, and another was taking over-the-counter supplements (e.g., biotin, melatonin, Vitamin D). They had all long stalled in terms of any improvements from pharmacologic or non-pharmacologic/psychotherapy interventions. All were treated at the Brain Wellness and Biofeedback Center of Washington (in Bethesda, Maryland, USA) in 20 individual FNS treatment sessions (except for one who moved after 17 sessions and who was doing well at the time of termination).

### FNS equipment and procedures

FNS consists of a laptop computer and J&J Enterprises (Poulsbo, WA) I-300 Compact 2 (C-2) Channel EEG module with on-board feedback generating power. It uses proprietary software to link the digital brainwave recording device (C-2 module) through the computer, which then sets the parameters for the C-2 module to emit pulsed EM stimulation [19]. The system returns a signal to the participant via conduction from the C-2 module, varying as a function of the detectable peak EEG frequency (but offset from it), thereby permitting strategic distortion of the EEG. The amount of EM stimulation was standardized with the feedback frequency being offset from the dominant EEG frequency at +20 Hertz (Hz). Pulses of EM energy operated at a duty cycle of 1 %, that is, of the maximum permissible on-time for each pulse, they were powered no more than 1 % of the time (e.g., the maximum on-time at 1 % for 1 Hz pulse was 0.01 s). Testing revealed a power level of 100 pico watts through the sensor cable (Weber Innovations, Ann Arbor, Michigan, USA).

Participants attended approximately 2–3 sessions per week. They sat comfortably with eyes closed and engaged in no specific activity. Electrodes were placed in a predetermined order over all areas of the cortex over the course of 20 sessions. Each session included a total of 4 s of EM stimulation spaced over 4 minutes. The stimulation was not immediately discernible and adverse reactions (e.g., transient increases in typical symptoms following the first few sessions) were minimal. Participants were not asked to discuss past traumas as part of the process.

### Measures

#### Brief Pain Inventory-Headache (BPI-HA) [20]

The Brief Pain Inventory was modified to indicate 0–10 numerical ratings specifically for pain intensity of headaches experienced in the past week (0 = no pain, 10 = pain as bad as you can imagine), including the worst headache pain and average headache pain during that period. This was completed at the outset before any receipt of the experimental intervention (i.e., beginning of treatment with session 1) and periodically thereafter including at sessions 5, 10, 15, and 20 (end of treatment).

#### Posttraumatic Stress Disorder Checklist-Military version (PCL-M) [21, 22]

The PCL-M is a widely used, highly reliable, and valid measure of the 17 symptoms (each rated on a 1 = not at all bothered to 5 = extremely bothered scale) comprising the typical diagnostic criteria for PTSD. It is specifically worded to reflect trauma experienced during military service. It was administered to derive a total score of PTSD severity (which can range from 17 to 85) at the beginning of treatment, session 1, and again at sessions 5, 10, 15, and 20 (end of treatment).

#### Cognitive clouding

A 0−10 numerical rating scale to assess extent of perceived cognitive dysfunction was completed by participants at the beginning of each of the 20 treatment sessions. “Cognitive clouding” was defined as problems with clearness of thinking, attention/concentration or memory problems; feeling “foggy,” “hazy,” or “clouded”; with 0 = no cognitive clouding and 10 = worst cognitive clouding possible.

### Data analysis

Data analyses included beginning (session 1) to end of treatment (session 20) t-test comparisons for BPI-HA worst and average headache pain ratings, beginning to end of treatment PCL-M total scores, and first treatment session to last treatment session 0–10 numerical ratings for subjective cognitive dysfunction.

## Results and discussion

All participants rated their worst headache during the past week to have been at least in the moderate range on the BPI-HA at the outset of treatment (moderate defined as ≥ 4 on 0–10 scale, with participants’ actual range being 5–9). Table 1 presents means and standard deviations for each of the measures at sessions 1, 5, 10, 15, and 20, along with t-test comparisons for beginning (session 1) to end of treatment (session 20) scores. Participants typically did not report bothersome side effects; however, a few (rarely) reported minor intensifications of their typical symptoms following a treatment session, which was then followed by a more marked reduction in symptom intensity. Progressive declines in symptom scores were generally observed across the various study time points. Highly significant reductions in headache pain were in evidence by the end of treatment for both the worst and average headache ratings, decreasing from moderate/severe levels to generally only mild levels. A review of individual score patterns indicated 3 participants reported no pain over the past week by end of treatment. In tandem with these improvements, posttraumatic stress symptoms and perceived cognitive dysfunction also decreased significantly from beginning to end of treatment. However, a review of individual score patterns indicated that one participant did not experience any reduction in headaches or other symptoms. Another did not experience any reduction in posttraumatic stress symptoms or perceived cognitive dysfunction; and, if anything, reported some minor worsening of these symptoms despite some modest improvement in headache ratings from moderately severe worst and average headache pain intensity to ratings in the mild to low moderate range. Medication usage, according to participants’ self-report, remained stable or decreased, although it was not possible to independently verify this with pharmacy or other records.

**Table 1:** The means and standard deviations (*M* ± *SD*) for measures at sessions 1 (beginning of treatment), 5, 10, 15, and 20 (end of treatment) as well as beginning to end of treatment *t*-test comparisons

Item	Session 1	Session 5	Session 10	Session 15	Session 20	*t* (df = 8)	Significance *P*	*r* ^*2*^
	Beginning of treatment				End of treatment			
BPI-HA worst pain past week	7.33 ± 1.22	4.78 ± 3.15	3.94 ± 2.83	3.28 ± 2.68	2.89 ± 2.62	5.43	0.001	0.79
BPI-HA average pain past week	4.56 ± 1.59	3.00 ± 2.83	2.56 ± 2.07	2.00 ± 2.00	1.39 ± 1.22	5.86	<0.001	0.81
PCL-M total score	48.56 ± 16.13	43.61 ± 15.02	41.50 ± 10.62	38.44 ± 12.81	33.39 ± 15.86	3.44	0.009	0.60
Cognitive clouding 0–10 NRS	7.22 ± 1.72	5.67 ± 1.12	5.22 ± 1.79	4.00 ± 1.32	3.72 ± 1.89	4.53	0.002	0.72

Effect size represented in *r* ^*2*^, percentage of variance explained

*BPI-HA* Brief Pain Inventory-Headache, *PCL-M* Posttraumatic Stress Disorder Checklist-Military version, *NRS* numerical rating scale

These findings suggest FNS may be a potentially useful and safe treatment for chronic daily headache following TBI, and that improvement in headache may also be associated in most persons with significant reductions in co-morbid posttraumatic stress symptoms and perceived cognitive dysfunction. However, contrary to the general trend, one of the nine participants in this study did not report any symptom improvement; and another did not report improvement in posttraumatic stress symptoms or cognitive dysfunction, although he did experience some reduction in headache intensity ratings down to low moderate/mild levels. Hence, it is likely that there are subgroups of patients with varying degrees of potential to respond to this intervention. Also, given the small sample size and lack of a randomized controlled clinical trial, we cannot rule out placebo responding or attention or other nonspecific effects contributing to the positive outcomes [23]. Further, the durability of effects is unknown, since we did not have extended follow-up information at this stage of the investigation. In addition, the manner in which chronic headache is influenced by or influences comorbid posttraumatic stress symptoms and features of cognitive dysfunction remains to be clarified. However, the findings provide preliminary support to justify future research within the design of a randomized, controlled clinical trial to more rigorously investigate the efficacy of FNS for treatment of posttraumatic headache and to better identify the characteristics of those most likely to respond favorably.

## Conclusions

Chronic headache remains a challenging and often vexing problem following TBI and which, particularly in military personnel with comorbid PTSD, may result in limited response to typical pharmacologic and non-pharmacologic treatments. Neurotherapy in the form of FNS as described here may be of potential efficacy in at least a subset of persons with persistent posttraumatic headache pain. Future research is warranted to better investigate the potential of FNS for symptom relief in terms of chronic headache pain; to examine the durability of any salutary effects; to identify the characteristics of those individuals most likely to obtain positive outcomes with FNS; to better understand the interactions with posttraumatic stress or other emotional dysfunction; and to explore the mechanisms underlying improvements across the domains of headache pain, cognitive dysfunction, and posttraumatic stress symptoms.
